# DeepGene-BC: Deep Learning-Based Breast Cancer Subtype Prediction via Somatic Point Mutation Profiles

**DOI:** 10.3390/cancers18040570

**Published:** 2026-02-09

**Authors:** Pengfei Hou, Liangjie Liu, Yijia Duan, Shanshan Yin, Wenqian Yan, Chongchen Pang, Yang Yan, Sabreena Aziz, Mika Torhola, Henna Kujanen, Klaus Förger, Hui Shi, Guang He, Yi Shi

**Affiliations:** 1 Bio-X Institutes, Key Laboratory for the Genetics of Developmental and Neuropsychiatric Disorders, Shanghai Jiao Tong University, Shanghai 200030, Chinaheguang@sjtu.edu.cn (G.H.); 2 Shanghai Key Laboratory of Psychotic Disorders, Brain Science and Technology Research Center, Shanghai Jiao Tong University, Shanghai 200030, China; 3 Shanghai Key Laboratory for Nucleic Acid Chemistry and Nanomedicine, Department of Laboratory Medicine, Institute of Molecular Medicine, Renji Hospital, School of Medicine, Shanghai Jiao Tong University, Shanghai 200127, China; 4 College of Chemistry and Materials Science, Shanghai Normal University, Shanghai 200233, China; 5 Atostek Oy, Hermiankatu 3 A, 33720 Tampere, Finland; 6 Department of Thoracic Surgery, Shanghai Chest Hospital, Shanghai Jiao Tong University, Shanghai 200025, China

**Keywords:** breast cancer, somatic mutation, cancer subtype, deep learning

## Abstract

**Background**: Molecular subtyping of breast cancer usually relies on transcriptomic profiles, a method constrained by limitations in robustness and clinical applicability. While somatic point mutations represent a stable genomic alternative, their predictive utility is hindered by high dimensionality, extreme sparsity, and weak single-gene associations. **Methods**: Here, we present deepGene-BC, a deep learning framework that synergizes a pathway-informed feature selection strategy with a hybrid neural network tailored for sparse binary data. To distill sparse genome-wide mutations into a compact and interpretable feature set, deepGene-BC integrates mutation recurrence filtering, curated pathway priors, and mutual information-based gene prioritization. These refined features are subsequently modeled using a specialized hybrid architecture designed to capture complex linear effects, feature interactions, and higher-order nonlinear patterns. **Results**: When benchmarked against an independent test set (*n* = 273) from the TCGA breast cancer cohort, deepGene-BC achieved an overall accuracy of 77.3% and an average sensitivity of 75.2%, accompanied by a strong overall discriminative performance (macro-averaged AU-ROC = 0.94, 95% CI: 0.92–0.96). **Conclusions**: By effectively combining biologically informed feature engineering with deep learning, deepGene-BC holds significant promise for non-invasive molecular stratification and precision oncology.

## Simple Summary

Breast cancer subtypes are critical for treatment selection and disease monitoring, but current classification methods rely on invasive tumor biopsies and transcriptomic assays that may not be suitable for repeated sampling. Somatic DNA mutations provide stable molecular markers that can be detected from circulating cell-free DNA, offering opportunities for minimally invasive tumor profiling. In this study, we explore the feasibility of using mutation profiles to infer breast cancer molecular subtypes. We propose deepGene-BC, a computational framework that extracts subtype-associated signals from sparse somatic mutation data. Using tissue-derived sequencing data as a proof of concept, we demonstrate that mutation patterns can recapitulate established transcriptome-defined subtypes. This work establishes a foundation for future development of mutation-based liquid biopsy approaches for longitudinal disease monitoring and precision oncology.

## 1. Introduction

Breast cancer is the most diagnosed malignancy and a leading cause of cancer-related mortality among women worldwide [1,2]. It is a biologically heterogeneous disease comprising multiple molecular subtypes with distinct clinical behaviors, prognoses, and therapeutic vulnerabilities [3,4,5,6]. Gene expression-based classification approaches, most notably the PAM50 classifier [7], stratify breast tumors into Luminal A, Luminal B, HER2-enriched, Basal-like, and Normal-like subtypes and are widely recognized in the area of clinical decision-making [8]. Despite their widespread clinical utility, transcriptome-based classifiers are constrained by several intrinsic limitations. First, reliance on mRNA profiling renders these methods highly sensitive to RNA degradation, batch effects, and pre-analytical variability. This issue is particularly exacerbated in routine formalin-fixed paraffin-embedded (FFPE) specimens, potentially compromising classification accuracy [9,10,11,12]. Furthermore, as PAM50 relies on invasive tissue sampling, it provides only a static, spatially confined snapshot of tumor biology at a single time point. This limitation is particularly relevant given the dynamic nature of breast cancer, in which molecular subtypes may evolve or transition over the course of disease progression or treatment, rendering subtype assignments based on primary tumor biopsies potentially discordant with the tumor’s current biological state [13,14,15].

Somatic point mutations represent a stable and fundamentally distinct layer of molecular information that complements transcriptomic data [16,17,18,19]. Unlike transient gene expression profiles, somatic mutations are cumulative events that directly record the evolutionary history of tumor cells [20,21,22]. More importantly, these mutational profiles can be readily detected from circulating cell-free DNA (cfDNA) via liquid biopsy, enabling minimally invasive early cancer detection and real-time disease monitoring [23,24,25,26]. In addition to diagnosis, cancer neoantigens derived from detected somatic mutations can serve as early treatment options [27,28].

Despite these advantages, leveraging somatic point mutation data for molecular subtype prediction or cancer diagnosis remains challenging. First, mutation profiles reside in a high-dimensional yet extremely sparse feature space, as each tumor or its subtype harbors mutations in only a limited number of genes, complicating effective feature representation and model training [29,30,31,32]. In addition, many functionally relevant alterations occur at low frequency and are therefore difficult to exploit using conventional recurrence-based or driver gene-focused approaches [33,34,35], which may discard informative signals beyond well-characterized cancer drivers. Finally, point mutations are discrete events whose associations with molecular phenotypes are often nonlinear and context-dependent [36,37], further limiting the effectiveness of standard feature selection and modeling strategies.

Here we present deepGene-BC, a deep learning approach for breast cancer molecular subtype prediction using somatic point mutation profiles from gene coding regions. It incorporates a pathway-constrained feature selection strategy that integrates mutation recurrence filtering, curated biological pathway priors, and mutual-information-based gene selection, thereby effectively condensing the vast genomic landscape into a compact, biologically interpretable feature set. These features are subsequently integrated into a neural network optimized for sparse binary tabular data, enabling the capture of both low- and high-order mutational interactions. Although deepGene-BC is motivated by the clinical promise of mutation-based liquid biopsy, the current study focuses on establishing a proof-of-concept using tissue-derived mutation profiles from TCGA, where tumor-derived signals are abundant and well-characterized. Benchmarked on the TCGA breast cancer cohort [38], deepGene-BC achieved robust classification across four PAM50 subtypes (Luminal A, Luminal B, HER2-enriched, and Basal-like), yielding a macro-averaged F1-score of 0.75 (95% CI: 0.689–0.807), an average sensitivity of 75.2%, an overall accuracy of 77.3%, and a strong macro-averaged AU-ROC of 0.94 (95% CI: 0.92–0.96), indicating balanced predictive performance across subtypes despite pronounced class imbalance and mutation sparsity. Notably, subtype-specific evaluation revealed improved discrimination for Basal-like (sensitivity = 79.6%) and HER2-enriched tumors (sensitivity = 69.6%), which are known to harbor more distinctive mutational patterns, whereas Luminal A and Luminal B subtypes exhibited moderate overlap, consistent with their biological similarity. These results demonstrate that stable genomic alterations encode sufficient signal to recapitulate transcriptome-defined taxonomies, offering a resilient alternative for clinical subtyping.

## 2. Materials and Methods

### 2.1. Mutation Data Process

Somatic mutation data (Mutation Annotation Format, MAF) for the TCGA breast cancer (BRCA) cohort were obtained from the Genomic Data Commons (GDC) portal. Data processing and visualization were performed using the R package maftools (version 2.16.0). To ensure the biological relevance of the input features, we focused on non-synonymous somatic variants that potentially impact protein function. The mutCountMatrix function within maftools was subsequently utilized to convert the filtered MAF data into a binary gene-sample mutation matrix, where 1 indicates the presence of at least one non-synonymous mutation in a given gene for a given sample and 0 indicates the wild-type status.

### 2.2. Mutual Information

To identify the most informative mutational feature within each pathway group, we computed the Mutual Information (MI) between the binary mutation status of each gene and the discrete target variable (molecular subtypes). Unlike correlation-based metrics (e.g., Pearson correlation), which only capture linear relationships, MI measures the reduction in uncertainty about one variable given knowledge of another, enabling the detection of both linear and non-linear dependencies between somatic mutations and tumor subtypes. Mathematically, for a discrete mutation feature X (where $x\;\in \;\left\{0,\;1\right\}$ represents wild-type or mutated status) and the discrete target variable Y (representing the molecular subtypes), the Mutual Information I(X; Y) is defined as

$$I(X;Y)=\underset{y\in Y}{\sum }\underset{x\in X}{\sum }p(x,y)log(\frac{p(x,y)}{p(x)p(y)})$$

### 2.3. Model Architecture

To effectively capture the complex mutational landscape of breast cancer, we designed a hybrid neural network architecture (Figure 4a) inspired by the DeepFM [39] framework. This architecture is specifically tailored for high-dimensional, sparse binary data, enabling the simultaneous modeling of three distinct types of molecular signals: (1) Linear effects from individual gene mutations, (2) Second-order feature interactions representing gene-gene co-occurrence or mutual exclusivity patterns, and (3) High-order non-linear associations characteristic of complex biological pathways. From a biological perspective, these three components can be intuitively interpreted as modeling single-gene driver effects, coordinated mutation patterns across genes, and pathway-level dysregulation underlying breast cancer subtypes.

The model takes the binary mutation vector $x\in {\left\{0,1\right\}}^{N}$ (where N=244) as input and processes it through three parallel components:

(1) The Linear (Wide) Component: A simple linear regression layer that models the direct contribution of each mutation to the subtype logits. It is defined as
  $$y_{\mathrm{linear}}=W_{\mathrm{linear}}x+b$$
  This component captures the marginal effect of individual gene mutations, which can reflect the influence of well-known subtype-associated driver genes (e.g., TP53, PIK3CA, or GATA3), as mutations in such genes alone may bias tumors toward specific molecular subtypes.
(2) The Factorization Machine (FM) Component: To capture pairwise gene interactions without the combinatorial explosion of parameters, we projected the binary input into a dense embedding space. Each feature i is assigned a latent vector $v_{i}\in R^{K}\;$(embedding dimension K=4). The FM component computes second-order interactions via the inner product of these latent vectors:
  $$y_{FM}=\sum_{i=1}^{N}\sum_{j=i+1}^{N}\langle v_{i},v_{j}\rangle x_{i}x_{j}$$
  Biologically, this branch models co-occurring or mutually exclusive mutation patterns, capturing gene–gene relationships that may reflect functional cooperation, synthetic lethality, or shared involvement in signaling pathways (e.g., coordinated alterations within the PI3K-AKT or cell-cycle regulatory axes).
(3) The Deep Component: To extract higher-order patterns, the feature embeddings are aggregated via sum-pooling and fed into a Multi-Layer Perceptron (MLP). The MLP consists of two hidden layers with 64 and 32 units, respectively. Each dense layer employs ReLU activation to introduce non-linearity and Dropout to prevent overfitting. The output is a vector $y_{deep}$ representing high-level semantic abstractions of the mutational profile. This component captures complex, non-linear interactions among multiple mutations simultaneously, which may correspond to pathway-level dysregulation and broader molecular programs that cannot be explained by individual genes or pairwise interactions alone.

The final prediction is obtained by summing the logits from all three components:

$$y_{\mathrm{total}}=y_{\mathrm{linear}}+y_{\mathrm{FM}}+y_{\mathrm{deep}}$$

By integrating signals at the single-gene, gene-pair, and pathway levels, the model offers an interpretable framework that facilitates biologically meaningful subtype prediction from sparse mutation data.

These combined logits are then passed through a Softmax function to generate the probability distribution over the four molecular subtypes.

### 2.4. Model Training

Model training was conducted using the PyTorch framework (version 2.7.0). Model parameters were optimized using the Adam optimizer, with default momentum parameters ($\beta_{1}$ = 0.9, $\beta_{2}$ = 0.999). The initial learning rate was set to 1 × 10^−3^ and decayed by a factor of 0.5 if the validation loss failed to improve for 10 consecutive epochs. To improve generalization, L2 regularization (weight decay = 1 × 10^−3^) was applied to the parameters of deep and linear layers only. Models were trained with a batch size of 64 for a maximum of 300 epochs.

To prevent overfitting, early stopping was applied based on validation performance. Training was terminated if the validation loss did not improve (threshold = 1 × 10^−3^) for 10 consecutive epochs, and the model parameters corresponding to the best validation loss were retained for downstream evaluation.

Given the inherent class imbalance among PAM50 breast cancer subtypes, we primarily addressed this issue at the evaluation stage by reporting macro-averaged performance metrics, which treat all classes equally regardless of sample size. During training, no dataset-level over- or under-sampling was performed. Instead, class imbalance was addressed through a sampling-based rebalancing strategy at the mini-batch level, where sample weights were defined as the inverse of class frequencies in the training set. This design increases the probability of sampling minority-class instances during stochastic optimization, thereby alleviating class imbalance effects without explicitly modifying the loss function or the underlying dataset distribution.

All hyperparameters were selected based on preliminary experiments on the training set and held constant across all experiments unless otherwise stated.

### 2.5. Cross Validation

To rigorously evaluate the performance of deepGene-BC and mitigate the risk of overfitting, we implemented a stratified 5-fold cross-validation strategy using the StratifiedKFold class from the scikit-learn library (version 1.8.0) in Python. The dataset was randomly partitioned into five non-overlapping folds, ensuring that the proportion of samples from each molecular subtype (Basal, HER2, LumA, and LumB) was preserved across all training and validation subsets. In each iteration, four folds were used for model training and hyperparameter tuning, while the remaining fold served as the internal validation set. This process was repeated five times such that every sample was used for validation exactly once. The final model performance was evaluated using accuracy, sensitivity (recall), specificity, precision, and the F1-score. For each metric, we reported the average value across the five validation folds to ensure a robust assessment. These metrics are mathematically defined as follows:

$$Sensitivity=\frac{TP}{TP+FN}$$

$$Specificity=\frac{TN}{TN+FP}$$

$$Precision=\frac{\;TP}{TP+FP}$$

$$Accuracy=\frac{TP+TN}{TP+TN+FP+FN}$$

$$F_{1}=\frac{2\times precision\times sensitivity}{precision+sensitivity}$$

### 2.6. Recurrence Threshold Selection and Sensitivity Analysis

To assess sensitivity to the mutation recurrence threshold, we evaluated multiple cutoff values, with the resulting gene sets summarized in Supplementary Table S1. Consistent with the long-tailed distribution of somatic mutations in breast cancer, the number of selected genes varied substantially across thresholds. Nevertheless, strong feature stability was observed: genes selected under stricter thresholds were largely retained at more permissive cutoffs, with inclusion rates ranging from 0.79 (1% vs. 0.5%) to 0.94 (0.25% vs. 0.1%). Pairwise Jaccard similarities between gene sets were orders of magnitude higher than expected under random selection. Feature prioritization was further assessed by Spearman rank correlation of mutual information-based gene rankings, comparing the top 71 genes (selected at the 1% threshold) across thresholds, which yielded near-perfect correlations (ρ ≈ 0.99). Together, these results indicate that the pathway-constrained mutual information framework reliably identifies a stable core set of informative genes, with looser thresholds primarily introducing additional low-frequency genes rather than altering the underlying feature structure.

Model performance across recurrence thresholds was evaluated and is reported in Supplementary Table S2. Performance remained stable across a broad range of thresholds. A stringent recurrence threshold of 1% selected only 71 genes and resulted in reduced performance, likely due to limited feature diversity and insufficient representational capacity. Relaxing the threshold to 0.5% and 0.25% increased the feature set to 244–426 genes and yielded consistently strong performance, suggesting that this range captures informative mutation patterns while maintaining an acceptable signal-to-noise ratio. In contrast, further lowering the threshold to 0.1% expanded the feature set to 626 genes but led to a modest performance decline, consistent with the inclusion of additional low-frequency, noise-prone mutations that contribute a limited discriminative signal.

### 2.7. Statistical Uncertainty Estimation by Bootstrap Resampling

To quantify statistical uncertainty in test-set performance, we applied a nonparametric bootstrap resampling procedure on the independent test cohort. Specifically, we generated 1000 bootstrap samples by resampling the test set, with each bootstrap sample having the same size as the original test set (*n* = 273). For each resampled dataset, model predictions were evaluated using the fixed trained model, and performance metrics, including accuracy, macro-averaged precision, recall, F1 score, and macro-averaged one-vs-rest AUC, were computed. For each metric, the empirical distribution across bootstrap replicates was used to estimate the mean and corresponding 95% confidence interval, defined by the 2.5th and 97.5th percentiles. This procedure provides a distribution-free estimate of performance variability and allows assessment of the stability of model performance on the held-out test set.

## 3. Results

### 3.1. Overview of deepGene-BC

**Figure 1:**
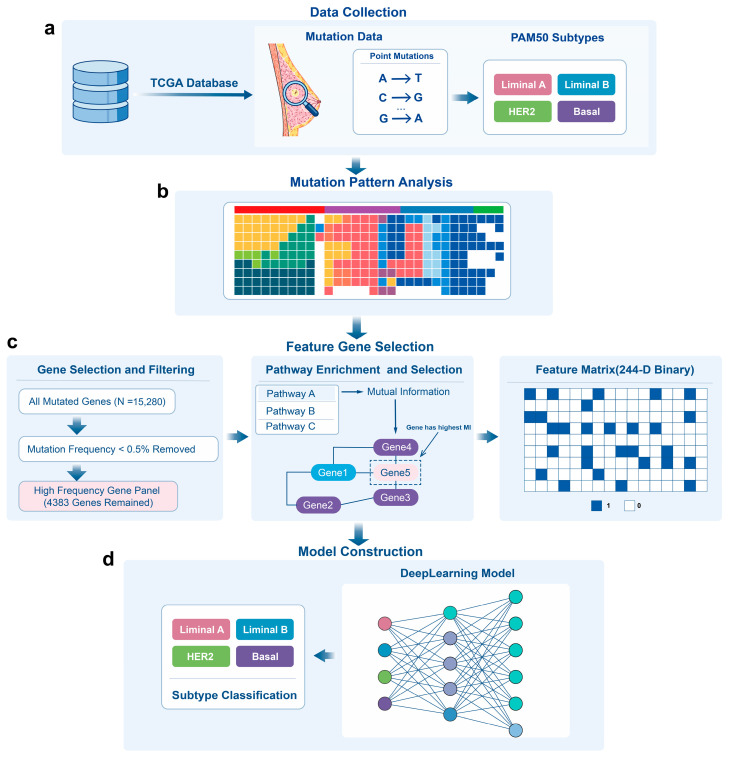
Overview of deepGene-BC. (**a**) Data acquisition: Integration of somatic point mutation profiles and corresponding clinical metadata from the TCGA-BRCA cohort. (**b**) Exploratory mutational analysis: Comparative analysis of mutation patterns across distinct molecular subtypes to identify subtype-specific genomic signatures, informing the subsequent feature selection. (**c**) Knowledge-guided feature engineering: A three-stage pipeline for robust feature gene selection and matrix construction: I. Filtering of genes with a mutation frequency below 0.5% to exclude stochastic background noise. II. Mapping genes to MSigDB C2 pathways and employing mutual information (MI) to rank gene-subtype associations. The gene with the maximal MI within each pathway is prioritized as a representative feature. III. Conversion of selected feature genes into a sparse binary mutation matrix for downstream modeling. (**d**) Implementation of a specialized neural network optimized for high-dimensional, sparse binary tabular data to capture both low-order and high-order mutational interactions.

deepGene-BC integrates large-scale somatic mutational landscapes with biologically constrained feature engineering and a neural network optimized for sparse binary inputs, enabling robust breast cancer subtype prediction based exclusively on somatic point mutations. As illustrated in Figure 1a, we collected somatic point mutation data and corresponding PAM50 subtype annotations from the TCGA breast cancer cohort. These mutation profiles were aggregated at the gene level to construct a binary mutation matrix indicating the presence or absence of at least one point mutation per gene in each sample. We next characterized global mutation patterns across tumors to quantify feature sparsity and inter-tumor heterogeneity, thereby highlighting the intrinsic challenges of mutation-based modeling (Figure 1b).

To address the high dimensionality and extreme sparsity of mutation features, deepGene-BC adopts a hierarchical feature selection strategy constrained by curated biological pathways (Figure 1c). First, genes were filtered based on mutation recurrence, retaining those mutated above a minimal frequency threshold of 0.5% to reduce the influence of ultra-rare events with limited statistical support. This step reduced the feature space from approximately 15,000 genes to about 4000 while preserving most recurrent mutational signals. Building on this filtered set, curated canonical pathways were leveraged as biological priors to organize genes into predefined pathway-based functional groups, rather than relying on data-driven clustering. Within each pathway, we computed the mutual information (MI) between individual gene mutation status and PAM50 subtype labels using the training set only and selected the gene with the highest MI as the representative feature for that pathway.

This pathway-constrained selection further condensed the feature space to a compact binary feature matrix comprising 244 pathway-representative genes. These low-dimensional, biologically informed sparse binary features were then used to train a deep learning classifier optimized for sparse high-dimensional tabular data (Figure 1d). The model outputs probabilistic predictions for the major PAM50 molecular subtypes (excluding the Normal-like subtype), enabling end-to-end subtype classification based solely on somatic point mutation profiles.

### 3.2. Sparsity and Heterogeneity of Somatic Point Mutations in Breast Cancer

**Figure 2:**
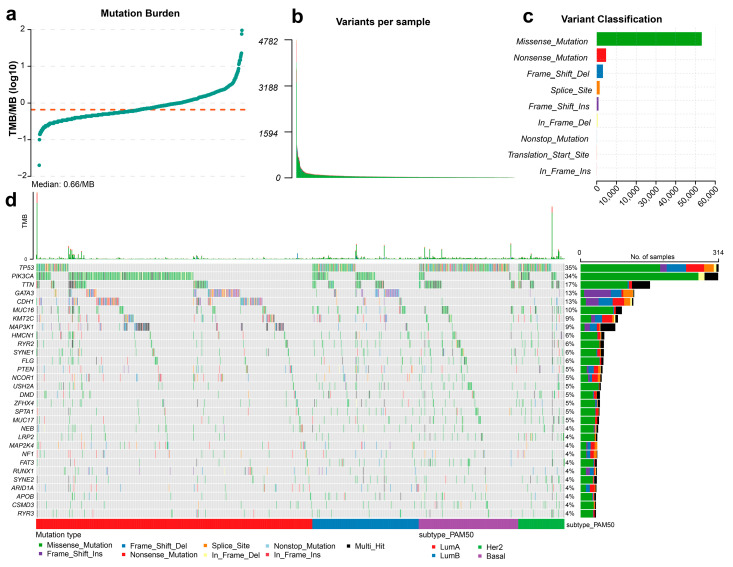
Global landscape, sparsity, and heterogeneity of somatic point mutations in breast cancer. (**a**) Distribution of tumor mutational burden (TMB) across samples, illustrating substantial inter-tumor variability. The red dashed line indicates the median mutation burden across all samples. (**b**) Distribution of the number of somatic variants per sample, revealing a highly skewed pattern, with most tumors harboring relatively few mutations. Somatic variant classes are distinguished by color, following the same color scheme as in panel (**c**). (**c**) Classification of somatic variants by functional consequence, highlighting the predominance of missense mutations. (**d**) Waterfall plot showing gene-level somatic point mutations across TCGA breast cancer samples, ordered by PAM50 molecular subtype. Frequently mutated genes are displayed on the left, with mutation types indicated by color. The upper panel shows tumor mutational burden (TMB) per sample.

We first examined somatic point mutation burden at the sample level across the TCGA breast cancer cohort. Overall, breast tumors exhibited relatively low to moderate tumor mutational burden (TMB), with a median of approximately 0.66 mutations per megabase (mut/Mb), whereas a small subset of tumors displayed markedly elevated mutation loads (Figure 2a). Consistent with this observation, the number of somatic variants per tumor spanned more than an order of magnitude, resulting in a highly skewed distribution in which most tumors harbored relatively few mutations (Figure 2b). Together, these results indicate pronounced inter-tumor heterogeneity in mutation burden among breast cancer samples. Beyond mutation burden, functional annotation of variants showed that mutations in breast cancer were predominantly missense substitutions, with other variant types occurring at substantially lower frequencies (Figure 2c and Supplementary Figure S1). At the gene level, mutation profiles were characterized by extreme sparsity and a pronounced long-tailed distribution: only a limited number of genes were recurrently mutated across the cohort, whereas most genes harbored mutations in only a small fraction of tumors (Figure 2d). Frequently mutated genes included well-established breast cancer drivers such as *TP53* and *PIK3CA*; however, even these alterations were present in only a subset of samples. This pattern indicates that most individual genes contribute limited marginal information when considered in isolation, posing a major challenge for mutation-based modeling strategies that rely on gene-level recurrence.

When stratified by PAM50 molecular subtype, mutation profiles exhibited subtype-associated tendencies while remaining largely probabilistic rather than deterministic. Basal-like tumors were enriched for *TP53* mutations, whereas Luminal subtypes showed relatively higher mutation frequencies in *PIK3CA* and other *PI3K* pathway-related genes (Figure 2d). Nevertheless, substantial overlap in mutation patterns was observed across subtypes, and no single mutation or small gene set was sufficient to uniquely define any PAM50 class. Together, these results demonstrate that subtype-relevant information is dispersed across numerous sparse and low-frequency mutations, underscoring the need for biologically informed strategies to extract meaningful signals from heterogeneous mutation landscapes.

### 3.3. Pathway-Constrained Extraction of Subtype-Associated Mutational Signals

**Figure 3:**
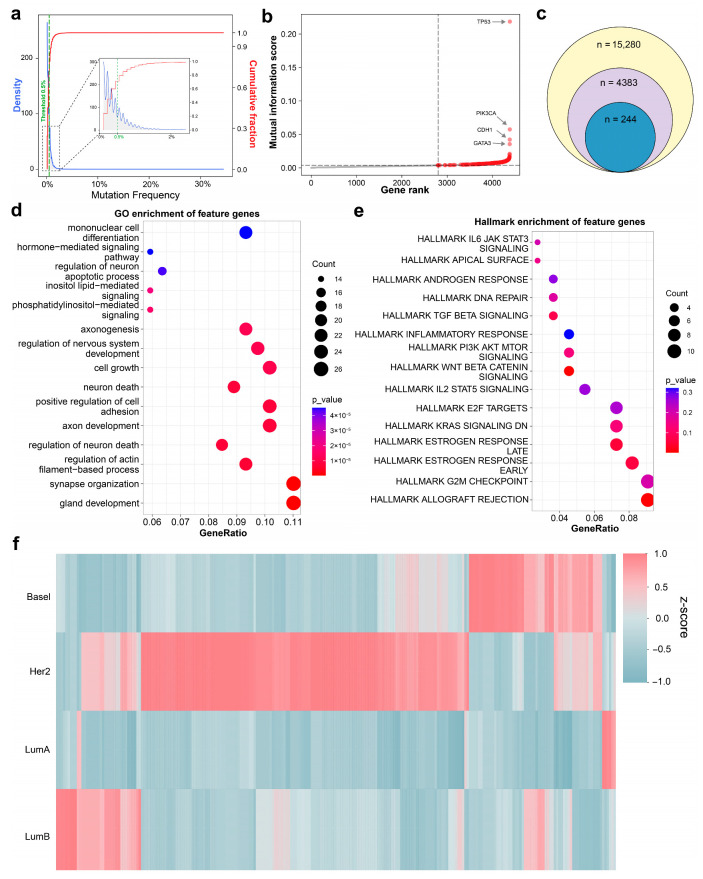
Pathway-constrained feature selection prioritizes biologically informative and subtype-discriminative genes. (**a**) Density plot (blue) and cumulative fraction (red) of somatic mutations across the cohort. The green dashed line indicates the 0.5% recurrence threshold used for initial noise reduction. (**b**) Distribution of MI scores between gene mutational status and PAM50 subtypes. High-impact drivers such as *TP53*, *PIK3CA*, and *GATA3* are highlighted as top-ranked features. (**c**) Venn diagram illustrating the systematic condensation of the feature space from 15,280 total genes to 4353 recurrently mutated genes and, finally, to 244 pathway-representative features. (**d**,**e**) Functional enrichment: Dot plots displaying (**d**) Gene Ontology (GO) and (**e**) MSigDB Hallmark pathway enrichment analysis of the 244 selected feature genes, adjusted by *p*-value (color) and gene ratio (size). (**f**) Heatmap showing the z-score normalized mutational distribution of the final 244 features across Basal-like (Basal), HER2-enriched (Her2), Luminal A (LumA), and Luminal B (LumB) subtypes.

To address the extreme sparsity and high dimensionality of somatic point mutation features, we first assessed gene-level mutation recurrence across the cohort. As expected, mutation frequencies followed a pronounced long-tailed distribution, with most genes mutated in only a small fraction of tumors (Figure 3a). Applying a minimum recurrence threshold of 0.5% effectively reduced the feature space from 15,280 to 4383 recurrently mutated genes while retaining most statistically supported mutational signals (Figure 3c, Section 2). A pathway-constrained feature selection strategy was then implemented to reduce redundancy while preserving biological interpretability, utilizing curated canonical pathways as structural priors. The cohort was randomly partitioned into training (70%) and testing (30%) sets, with all feature selection steps strictly confined to the training data to prevent information leakage. Genes passing the recurrence filter were assigned to MSigDB C2 pathway groups [40], and within each pathway, the gene exhibiting the highest mutual information (MI) with PAM50 subtype labels was selected as the representative feature. This procedure yielded a compact and biologically grounded feature set comprising 244 pathway-representative genes (Figure 3c). Across all genes, MI scores spanned a broad range, reflecting predominantly weak and heterogeneous associations between individual mutations and molecular subtypes (Figure 3b). Nevertheless, the selected features were enriched among top-ranked genes, including well-established breast cancer drivers such as *TP53*, *PIK3CA*, and *GATA3*, while also retaining lower-frequency genes with substantial subtype-associated information. Importantly, repeating the feature selection procedure using an alternative data split (30% training and 70% test) yielded a highly overlapping set of selected genes, indicating that the pathway-constrained and mutual information selection strategy is stable and robust to variations in training sample size (Supplementary Figure S2).

To assess the biological relevance of the 244 selected features, we performed functional enrichment analysis, which revealed a significant overrepresentation of canonical cancer-related biological processes. Gene Ontology (GO) analysis [41] highlighted fundamental pathways such as cell cycle regulation, DNA damage response, and signal transduction. Concurrently, MSigDB Hallmark enrichment pinpointed key oncogenic programs such as PI3K-AKT signaling and epithelial-mesenchymal transition (EMT) (Figure 3d,e). These findings indicate that the pathway-constrained selection framework preferentially captures biologically meaningful mutational features rather than a random gene subset. Beyond their collective biological relevance, these mutation features also exhibited distinct subtype-specific patterns. As shown in the heatmap of Z-score normalized mutation profiles, the features presented characteristic signatures across Basal-like, HER2-enriched, Luminal A, and Luminal B tumors (Figure 3f). This clear stratification confirms that our selected features effectively distill subtype-discriminative information from sparse somatic mutation data. Accordingly, this biologically informed and compact feature matrix was subsequently used as the input for downstream mutation-based subtype modeling.

### 3.4. deepGene-BC Architecture and Cross-Validation Performance

**Figure 4:**
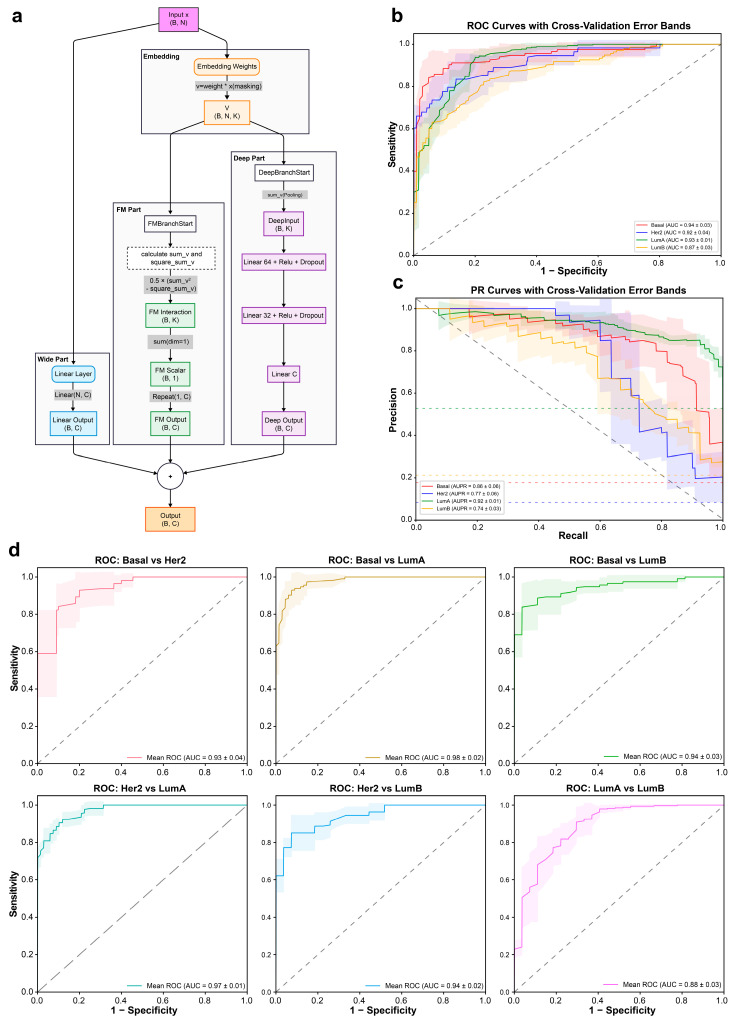
Architecture of deepGene-BC and cross-validation performance. (**a**) Overview of the deepGene-BC model architecture. The model integrates three parallel branches: a Wide branch for capturing linear feature patterns, a Factorization Machine (FM) branch for modeling pairwise feature interactions, and a Deep branch for learning higher-order nonlinear representations. Outputs from all branches are combined to generate final subtype classification probabilities. The symbol * denotes Hadamard product. (**b**) Multi-class receiver operating characteristic (ROC) curves for four breast cancer subtypes evaluated by cross-validation. Solid lines indicate mean ROC curves across folds and shaded regions denote standard deviation. (**c**) Precision–recall (PR) curves for the four subtypes under cross-validation. Solid lines represent mean PR curves across folds, with shaded regions indicating variability. (**d**) Pairwise ROC curves for all six subtype comparisons, summarized across cross-validation folds.

To effectively model sparse, high-dimensional somatic mutation profiles, we designed deepGene-BC as a hybrid deep learning framework that integrates complementary modeling paradigms specifically tailored to tabular binary data (Figure 4a). The architecture consists of three parallel components: a Wide branch that captures linear additive effects of individual mutations, a factorization-based branch that explicitly models second-order feature interactions, and a Deep branch that learns higher-order nonlinear representations through stacked fully connected layers. By aggregating these components, deepGene-BC jointly models marginal effects, pairwise interactions, and complex nonlinear dependencies among mutation features, enabling robust subtype inference from sparse mutation inputs.

We evaluated deepGene-BC using cross-validation on the TCGA breast cancer cohort, assessing its performance in multi-class classification across four PAM50 subtypes. Receiver operating characteristic (ROC) and precision–recall (PR) analyses demonstrated consistently strong discriminative performance across subtypes (Figure 4b,c). deepGene-BC achieved high discriminative performance across subtypes, with area under the ROC curve (AUC) values ranging from 0.87 to 0.94 and area under the PR curve (AUPR) values ranging from 0.74 to 0.92, demonstrating both strong sensitivity and robustness under class imbalance. The relatively narrow standard deviation bands across folds indicate stable and reproducible performance. PR analyses further highlighted the robustness of deepGene-BC for Basal-like and Luminal subtypes, underscoring its effectiveness in extracting subtype-specific mutational signals from sparse binary features. To further interrogate fine-grained subtype separability, we conducted pairwise (one-vs-one) ROC analyses across all six subtype combinations (Figure 4d). deepGene-BC exhibited strong discriminative performance for most subtype pairs, with particularly pronounced separability observed between Basal-like and Luminal tumors, as well as between HER2-enriched and Luminal A subtypes. In contrast, discrimination between Luminal A and Luminal B tumors was comparatively less distinct, likely reflecting their shared luminal lineage and overlapping biological programs. Together, these results suggest that somatic point mutation profiles capture informative subtype-associated signals, while also highlighting inherent biological constraints in resolving closely related subtypes solely based on mutation data.

Finally, we benchmarked deepGene-BC against several widely used machine learning and deep learning methods, including standard deep neural networks (DNNs, Supplementary Figure S3), support vector machines (SVMs, Supplementary Figure S4), random forests (RFs, Supplementary Figure S5), and k-nearest neighbors (KNNs, Supplementary Figure S6), using identical input features and cross-validation settings (Table 1). Across all evaluated metrics, deepGene-BC consistently outperformed the competing approaches, achieving an overall accuracy of 0.81 and a macro-averaged F1-score of 0.77. Together, these results highlight the advantage of deepGene-BC’s architecture in effectively leveraging sparse mutational data and demonstrate that explicit modeling of feature interactions and nonlinear effects is critical for accurate mutation-based breast cancer subtype classification.

### 3.5. Performance of deepGene-BC on the Independent Test Set

**Figure 5:**
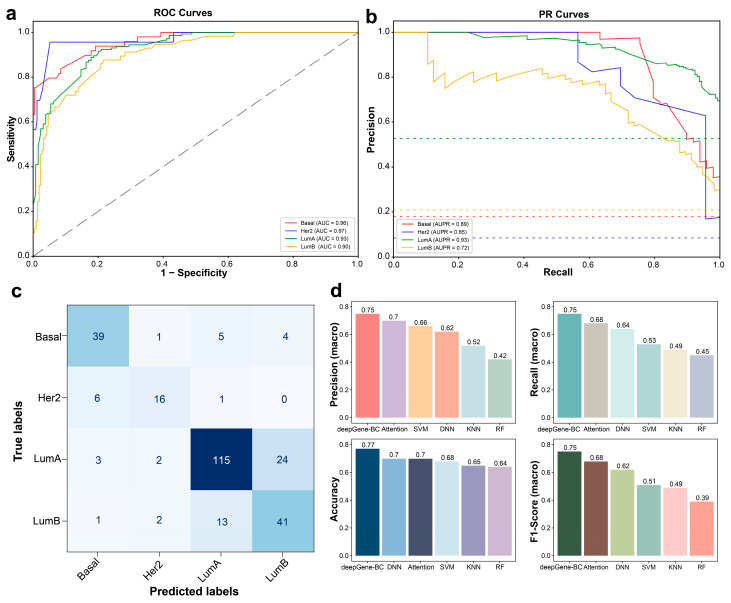
Independent test set performance of deepGene-BC. (**a**) One-vs-Rest ROC curves for four breast cancer subtypes evaluated on the held-out test set. (**b**) Precision–recall (PR) curves for the four subtypes on the test set. Dashed horizontal lines indicate the baseline precision corresponding to subtype prevalence. (**c**) Confusion matrix summarizing multi-class classification results on the independent test set. Darker colors indicate higher values in the matrix. (**d**) Comparison of deepGene-BC with alternative machine learning models on the same test set, evaluated using macro-precision, macro-recall, accuracy, and macro-F1.

We next evaluated the generalization performance of deepGene-BC on an independent held-out test set. ROC analysis showed strong discriminative performance across all four breast cancer subtypes, with One-vs-Rest AUC values of 0.96 for Basal-like, 0.97 for HER2-enriched, 0.93 for Luminal A, and 0.90 for Luminal B tumors (Figure 5a). Consistently, precision–recall (PR) curves showed robust precision–recall trade-offs despite subtype imbalance, with particularly high average precision observed for Basal-like and Luminal A subtypes (Figure 5b), indicating that deepGene-BC effectively captures subtype-specific mutation patterns in unseen samples. The confusion matrix further revealed detailed prediction behaviors of deepGene-BC on the test set (Figure 5c). Correct predictions were predominantly concentrated along the diagonal, reflecting accurate subtype assignment for most samples. Misclassifications occurred predominantly between Luminal A and Luminal B tumors, which is biologically plausible given their shared luminal characteristics and overlapping molecular features, whereas Basal-like tumors were more distinctly separated from other subtypes (Supplementary Figure S7). We next compared deepGene-BC with several commonly used machine learning and deep learning models, including a self-attention-based method (Attention), support vector machine (SVM), deep neural network (DNN), k-nearest neighbor (KNN), and random forest (RF), using the same test set and identical input features (Figure 5d). deepGene-BC consistently outperformed all competing methods across multiple evaluation metrics, achieving the highest precision (0.75), recall (0.75), accuracy (0.77), and F1-score (0.75). Notably, the superior F1-score highlights the balanced performance of deepGene-BC in jointly optimizing sensitivity and precision, underscoring its robustness and suitability for mutation-based multi-class breast cancer subtype classification. To ensure that this superior performance stems from biologically meaningful patterns rather than spurious correlations, we further examined the model’s interpretability. A detailed post-hoc feature importance analysis, which reveals how deepGene-BC captures canonical lineage drivers (e.g., *GATA3*, *TP53*) and genomic instability features, is provided in Supplementary Figure S8 and Supplementary Note S1.

### 3.6. Ablation Analysis of Model Architecture

To quantitatively assess the contribution of individual architectural components in deepGene-BC, we conducted an ablation analysis by evaluating several simplified variants of the model on the same independent test set. Specifically, we compared models using only the linear (Wide) component, only the factorization machine (FM) component, only the deep neural network (Deep) component, a combined Wide + Deep model, and the full Wide + FM + Deep architecture. All ablation models were trained and evaluated using identical data splits and hyperparameters.

As summarized in Supplementary Table S3, all ablated variants exhibited reduced performance compared with the full model, indicating that no single component alone was sufficient to capture the complexity of mutation-based subtype discrimination. The Wide-only model showed limited predictive capacity (macro-F1 = 0.41), reflecting the restricted expressiveness of linear mutation effects. The FM-only and Deep-only models achieved moderate performance, suggesting that pairwise interactions and higher-order nonlinear patterns each provide useful but incomplete information when modeled in isolation (FM-only: macro-F1 = 0.59, Deep-only: macro-F1 = 0.53). Importantly, combining components led to consistent performance gains. The Wide + Deep model outperformed all single-branch variants, highlighting the complementarity between linear effects and nonlinear representations (macro-F1 = 0.62). The full Wide + FM + Deep architecture achieved the strongest overall performance across all evaluated metrics (macro-F1 = 0.75), demonstrating that jointly modeling individual mutation effects, pairwise mutation interactions, and higher-order nonlinear associations yields the most effective representation for breast cancer subtype prediction.

## 4. Discussion

In this study, we present deepGene-BC, a deep learning method leveraging a pathway-informed feature selection strategy to address the intrinsic challenges of high dimensionality, sparsity, and weak single-gene signals characteristic of somatic point mutation data in breast cancer. By systematically characterizing the long-tailed distribution of mutation recurrence and integrating curated biological pathways as structural priors, deepGene-BC effectively distills subtype-associated mutational signals into a compact and biologically interpretable feature set. These sparse binary features are subsequently modeled using a hybrid neural architecture specifically optimized for mutation data, which jointly captures linear effects, pairwise feature interactions, and higher-order nonlinear patterns. This combined strategy enables robust learning from ultra-sparse mutation profiles while mitigating noise and redundancy inherent to genome-wide mutation data. Through comprehensive cross-validation and independent test set evaluations, deepGene-BC demonstrates strong and consistent performance in breast cancer subtype classification, outperforming conventional machine learning and deep learning baselines across multiple metrics. Importantly, the selected features are enriched for known breast cancer driver genes while also capturing informative lower-frequency mutations, underscoring the biological relevance and stability of the proposed feature selection strategy. Together, these results highlight the effectiveness of combining pathway-level biological constraints with flexible deep learning architectures for mutation-based cancer subtype inference.

Despite these strengths, several limitations of the current study warrant consideration. First, deepGene-BC is currently built exclusively on somatic point mutation presence and does not incorporate other layers of genomic regulation. In particular, the reduced separability between closely related subtypes such as Luminal A and Luminal B suggests that complementary information, including copy number alterations or mutation functional impact annotations, may provide additional discriminatory power. Incorporating such features represents a natural direction for future extensions of the framework, but is beyond the scope of the present study. In addition, while the current feature set achieves strong predictive performance, further refinement toward a smaller, optimized mutation panel may facilitate practical clinical applications. Deriving a minimal yet informative gene panel from deepGene-BC could enable cost-effective and sensitive subtype prediction using circulating cell-free DNA (cfDNA), thereby supporting non-invasive molecular stratification and disease monitoring. Beyond feature representation and model design, data source and cohort characteristics also play a critical role in determining model generalizability.

From a methodological perspective, tissue-derived sequencing data offer a controlled and well-annotated benchmark for evaluating mutation-based subtype inference, and thus represent a necessary proof-of-concept stage before addressing the additional complexity introduced by external cohorts, clinical sequencing panels, or liquid biopsy data. Nevertheless, translating mutation-based subtype classifiers from tissue-derived sequencing data cfDNA introduces nontrivial domain shift challenges. Compared with bulk tumor tissue, cfDNA samples are characterized by substantially lower and variable tumor fractions, heterogeneous sequencing coverage, and increased stochastic noise in variant allele frequencies. As a result, mutation profiles derived from cfDNA are often incomplete and noisier, with many alterations present at low allelic fractions or falling below detection thresholds. Consequently, models trained on tissue-derived mutation profiles, including deepGene-BC, are not expected to directly generalize to cfDNA data or other external sequencing settings without adaptation. Instead, deepGene-BC provides a principled starting point that may serve as a transferable modeling backbone, which can be recalibrated and fine-tuned using target-domain-specific training data when available. Such adaptation-based strategies have the potential to improve data efficiency relative to training models entirely from scratch, although systematic validation in cfDNA and multi-platform cohorts remains an important direction for future work.

## 5. Conclusions

In summary, deepGene-BC provides a scalable and interpretable framework for leveraging sparse somatic mutation data in cancer subtyping. By combining biologically grounded feature engineering with a flexible deep learning architecture, it establishes a promising foundation for future multi-omics integration and translational applications in precision oncology.

## Figures and Tables

**Table 1:** Cross-validation performance of deepGene-BC and many other deep learning and machine learning methods.

	deepGene-BC	DNN	SVM	KNN	RF
Accuracy	0.81 (±0.03)	0.73 (±0.03)	0.67 (±0.02)	0.66 (±0.04)	0.65 (±0.02)
Precision (macro)	0.81 (±0.04)	0.64 (±0.04)	0.56 (±0.04)	0.56 (±0.13)	0.42 (±0.08)
Recall (macro)	0.75 (±0.03)	0.63 (±0.04)	0.51 (±0.03)	0.48 (±0.06)	0.46 (±0.03)
F1 (macro)	0.77 (±0.04)	0.63 (±0.04)	0.52 (±0.03)	0.46 (±0.07)	0.42 (±0.04)

## Data Availability

The main data supporting the results are available within this article as well as its Supplementary Information. All the datasets adopted in this study are publicly available. The DNA methylation profiles of normal tissues adopted in this study were collected from the TCGA breast cancer cohort [https://portal.gdc.cancer.gov]. Code for the work in this manuscript is available on GitHub at https://github.com/jouhpf/deepGene-BC (accessed on 1 January 2026).

## Supplementary Materials

The following supporting information can be downloaded at https://www.mdpi.com/article/10.3390/cancers18040570/s1, Figure S1: Global mutational characteristics of breast cancer; Figure S2: Stability of mutual information–based feature selection across different data partitions; Figure S3: Cross-validation performance of the MLP model; Figure S4: Cross-validation performance of the SVM model; Figure S5: Cross-validation performance of the RF model; Figure S6: Cross-validation performance of the KNN model; Figure S7: Pairwise classification performance across breast cancer molecular subtypes on the independent test set; Figure S8: Feature importance analysis of deepGene-BC; Table S1: Quantitative stability analysis of feature genes identified across varying selection thresholds; Table S2: Predictive performance and robustness of deepGene-BC across different feature selection thresholds; Table S3: Ablation study of model components of deepGene-BC; Note S1: Model interpretability. References [42,43,44,45,46,47,48] are cited in the Supplementary Materials.
