# Supplementary material for: DeepGene-BC: Deep Learning-Based Breast Cancer Subtype Prediction via Somatic Point Mutation Profiles
Source: Cancers (Basel). 2026 Feb 9;18(4):570. doi: 10.3390/cancers18040570 (PMC12939208; doi:10.3390/cancers18040570)
Supplement: Supplementary file 1 [file cancers-18-00570-s001.zip › cancers-4113391-supplementary.pdf]

Supplementary Information

DeepGene-BC: Deep Learning-Based Breast Cancer Subtype Prediction via Somatic Point Mutation Profiles

Pengfei Hou [1,2,3,4,†], Liangjie Liu [1,2,†], Yijia Duan [1,2], Shanshan Yin [1,2], Wenqian Yan [1,2], Chongchen Pang [1,2], Yang Yan [1,2], Sabreena Aziz [1,2], Mika Torhola [5], Henna Kujanen [5], Klaus Förger [5], Hui Shi [6,*], Guang He [1,2] and Yi Shi [1,2,*]

[1] Bio-X Institutes, Key Laboratory for the Genetics of Developmental and Neuropsychiatric Disorders, Shanghai Jiao Tong University, Shanghai 200030, China

[2] Shanghai Key Laboratory of Psychotic Disorders, Brain Science and Technology Research Center, Shanghai Jiao Tong University, Shanghai 200030, China

[3] Institute of Molecular Medicine, Department of Laboratory Medicine, Shanghai Key Laboratory for Nucleic Acid Chemistry and Nanomedicine, Renji Hospital, School of Medicine, Shanghai Jiao Tong University, Shanghai 200127, China

[4] College of Chemistry and Materials Science, Shanghai Normal University, Shanghai 200233, China

[5] Atostek Oy, Hermiankatu 3 A, 33720 Tampere, Finland

[6] Department of Thoracic Surgery, Shanghai Chest Hospital, Shanghai Jiao Tong University, Shanghai 200025, China

***** Correspondence: disney1982@163.com (H.S.); yishi@sjtu.edu.cn (Y.S.)
† These authors contributed equally to this work.

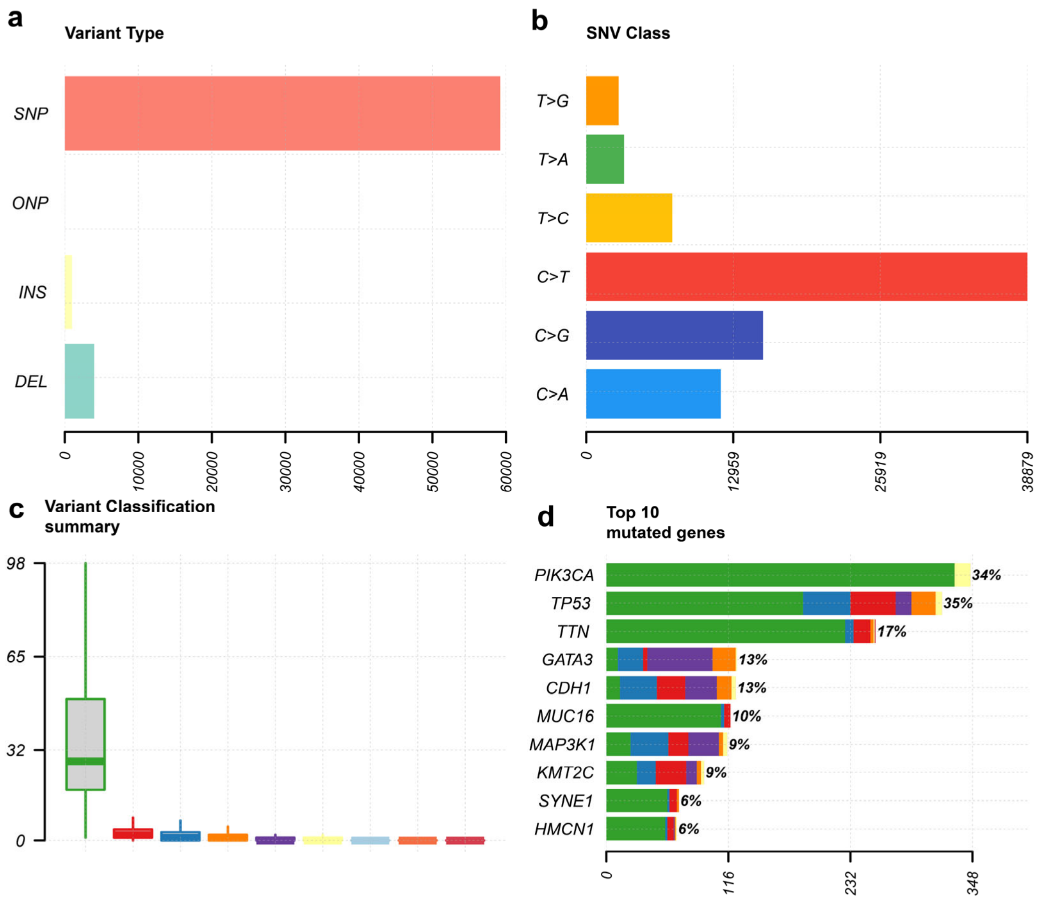

Supplementary Figure S1. Global mutational characteristics of breast cancer. a, Distribution of variant types across the cohort, showing that single-nucleotide polymorphisms (SNPs) constitute the predominant class of somatic variants, compared with insertions (INS), deletions (DEL), and oligonucleotide polymorphisms (ONPs). **b**, Spectrum of single-nucleotide variant (SNV) substitution classes, highlighting the dominance of C>T transitions. **c**, Distribution of functional variant classifications per sample, illustrating inter-tumor variability in mutation composition. **d**, Top 10 most frequently mutated genes in the cohort, ranked by mutation frequency. Bars are stacked and colored according to variant type.

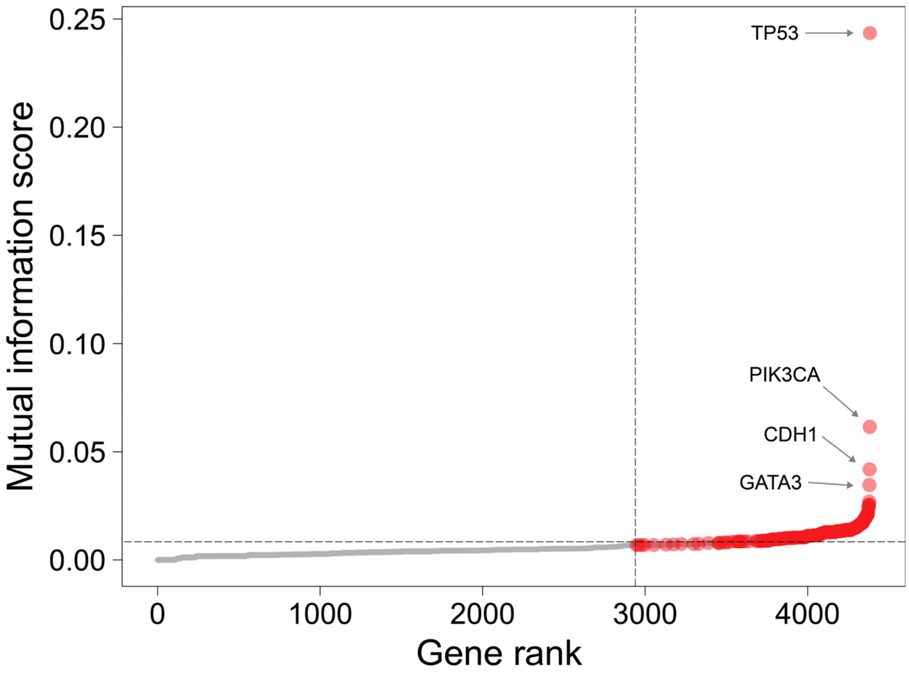

Supplementary Figure S2. Stability of mutual information–based feature selection across different data partitions. The distribution of mutual information (MI) scores between gene mutation status and PAM50 subtype labels is shown for an alternative random partition of the cohort into 30% training and 70% testing sets.

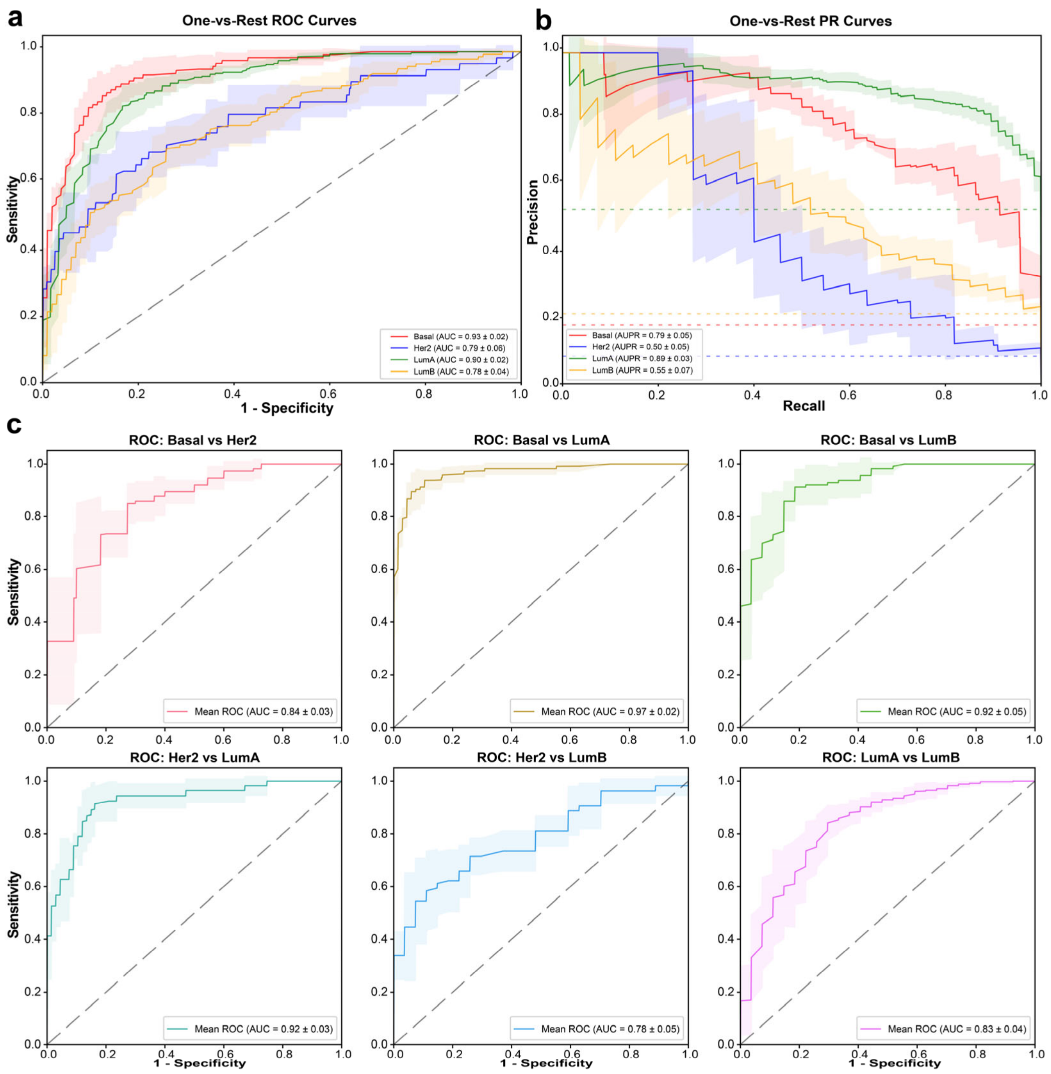

Supplementary Figure S3. Cross-validation performance of the MLP model. a, Receiver operating characteristic (ROC) curves for multi-class classification across four breast cancer subtypes. Solid lines represent the mean ROC curves across cross-validation folds, while shaded regions indicate the corresponding standard deviation. Mean area under the ROC curve (AUC) values with associated variability are annotated for each subtype. **b**, Precision-recall (PR) curves for multi-class classification across four breast cancer subtypes. Solid lines denote the mean PR curves across cross-validation folds, with shaded regions indicating the standard deviation. Mean area under the precision-recall curve (AUPR) values are annotated for each subtype. **c**, Pairwise ROC for all six subtype combinations. Solid lines represent the mean ROC curves across cross-validation folds, while shaded regions indicate the corresponding standard deviation.

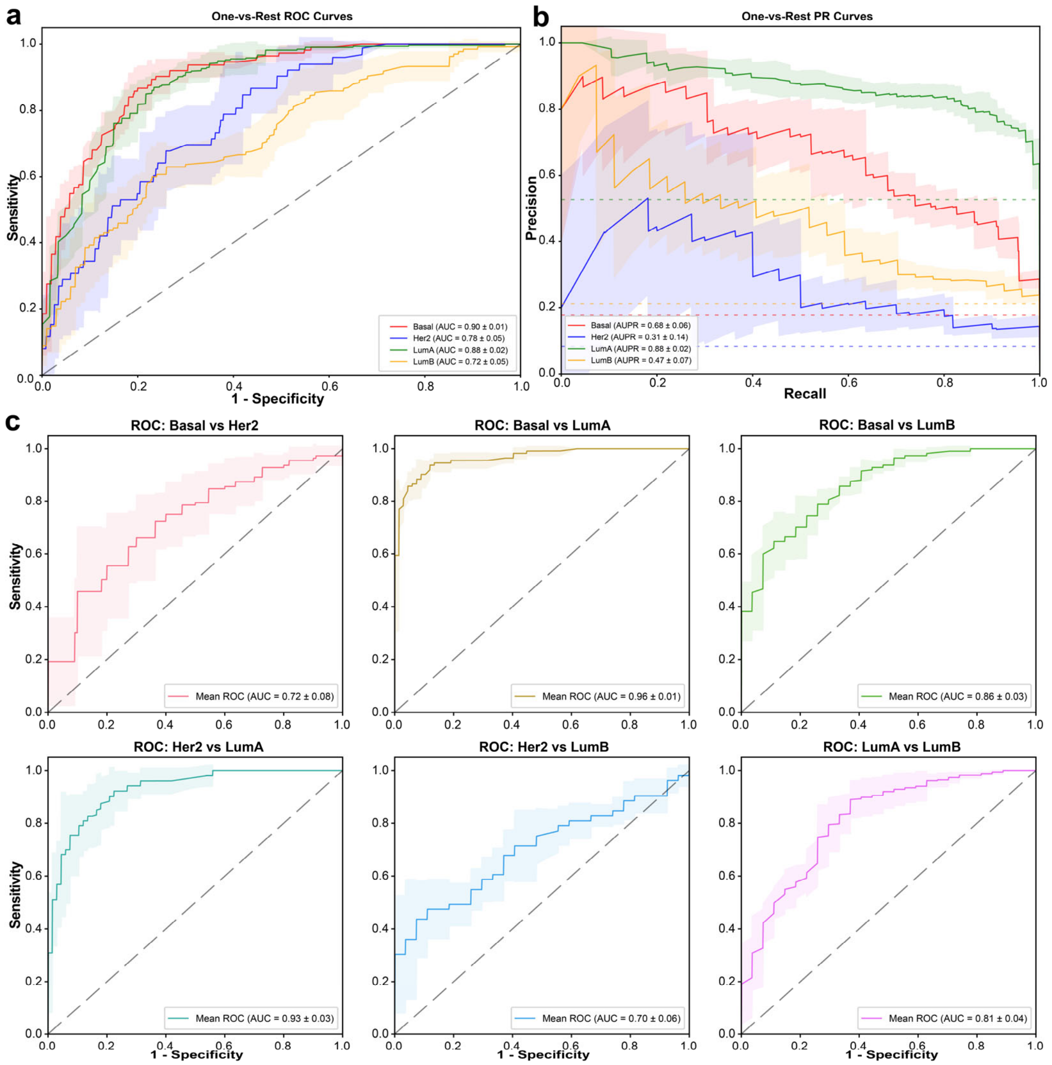

Supplementary Figure S4. Cross-validation performance of the SVM model. a, ROC curves for multi-class classification across four breast cancer subtypes. Solid lines represent the mean ROC curves across cross-validation folds, while shaded regions indicate the corresponding standard deviation. Mean AUC values with associated variability are annotated for each subtype. **b**, PR curves for multi-class classification across four breast cancer subtypes. Solid lines denote the mean PR curves across cross-validation folds, with shaded regions indicating the standard deviation. Mean AUPR values are annotated for each subtype. **c**, Pairwise ROC for all six subtype combinations. Solid lines represent the mean ROC curves across cross-validation folds, while shaded regions indicate the corresponding standard deviation.

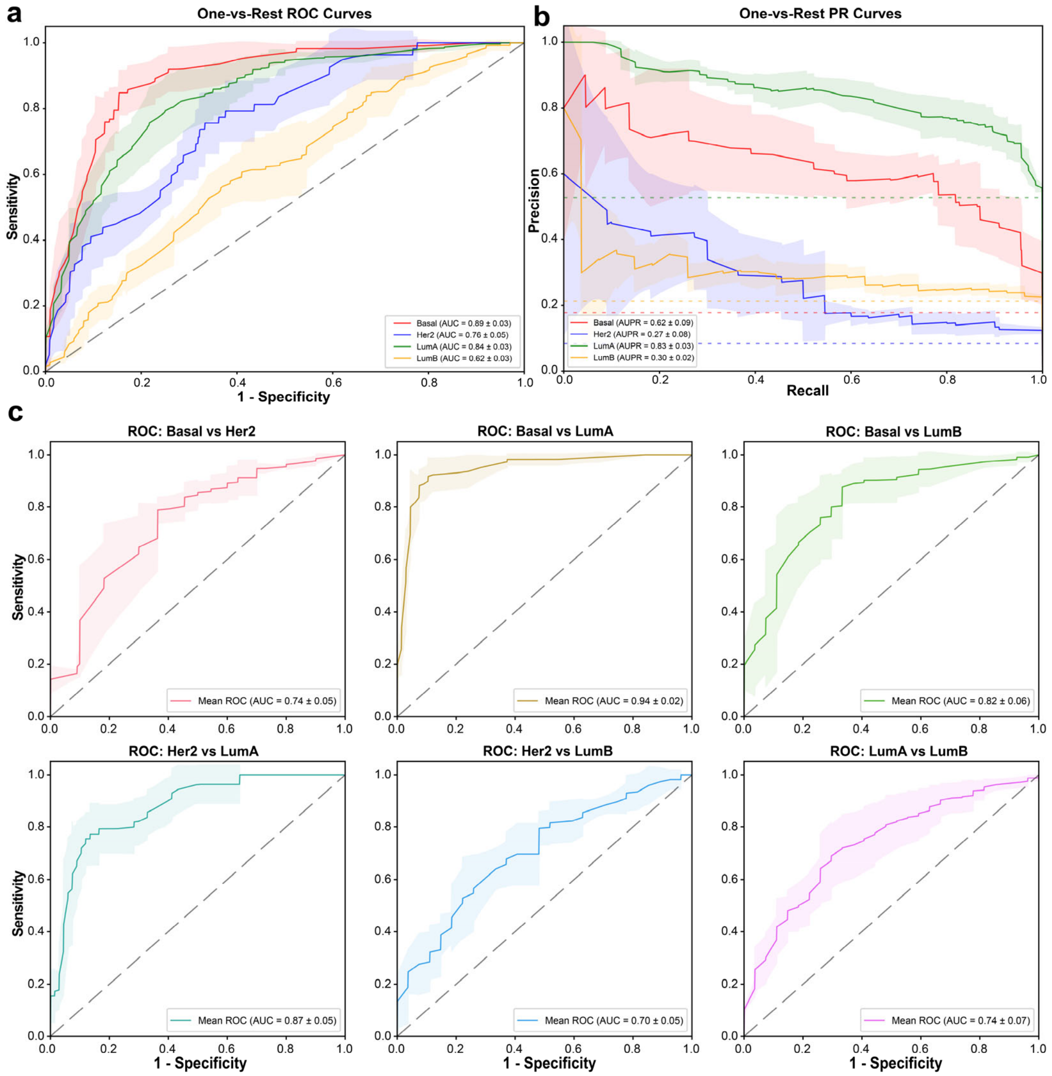

Supplementary Figure S5. Cross-validation performance of the RF model. a, ROC curves for multi-class classification across four breast cancer subtypes. Solid lines represent the mean ROC curves across cross-validation folds, while shaded regions indicate the corresponding standard deviation. Mean AUC values with associated variability are annotated for each subtype. **b**, PR curves for multi-class classification across four breast cancer subtypes. Solid lines denote the mean PR curves across cross-validation folds, with shaded regions indicating the standard deviation. Mean AUPR values are annotated for each subtype. **c**, Pairwise ROC for all six subtype combinations. Solid lines represent the mean ROC curves across cross-validation folds, while shaded regions indicate the corresponding standard deviation.

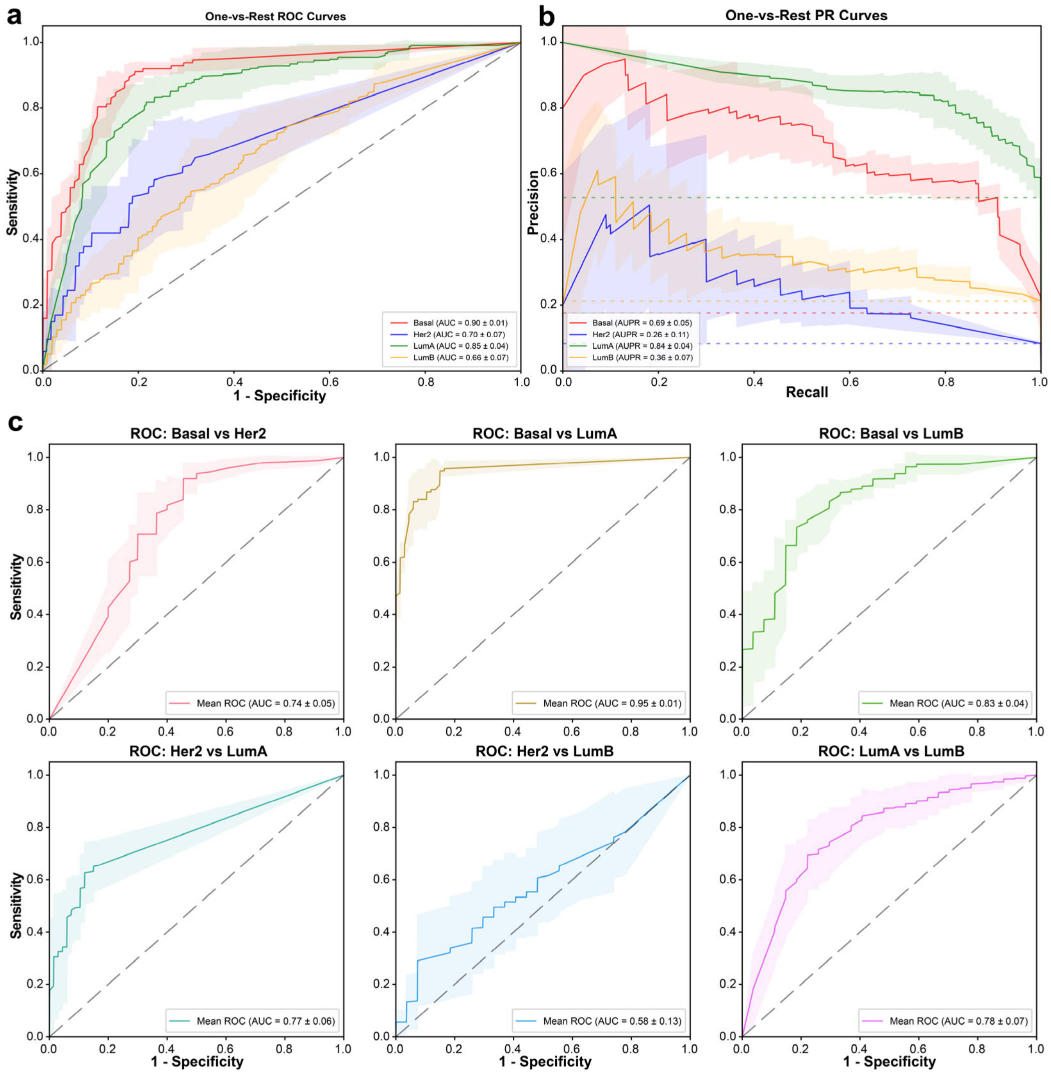

Supplementary Figure S6. Cross-validation performance of the KNN model. a, ROC curves for multi-class classification across four breast cancer subtypes. Solid lines represent the mean ROC curves across cross-validation folds, while shaded regions indicate the corresponding standard deviation. Mean AUC values with associated variability are annotated for each subtype. **b**, PR curves for multi-class classification across four breast cancer subtypes. Solid lines denote the mean PR curves across cross-validation folds, with shaded regions indicating the standard deviation. Mean AUPR values are annotated for each subtype. **c**, Pairwise ROC for all six subtype combinations. Solid lines represent the mean ROC curves across cross-validation folds, while shaded regions indicate the corresponding standard deviation.

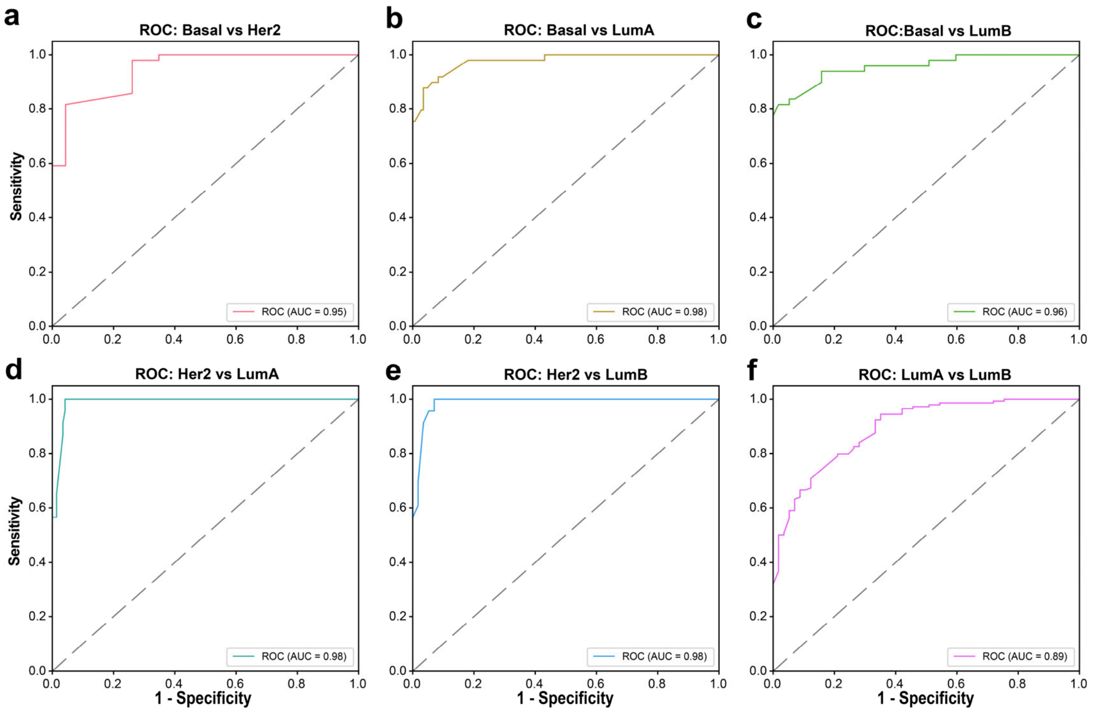

Supplementary Figure S7. Pairwise classification performance across breast cancer molecular subtypes on the independent test set. Evaluation on the independent test set across six one-vs-one combinations (**a-f**). AUC values are provided for each comparison, showing high discriminative power across most subtypes, with the lowest performance observed between Luminal A (LumA) and Luminal B (LumB).

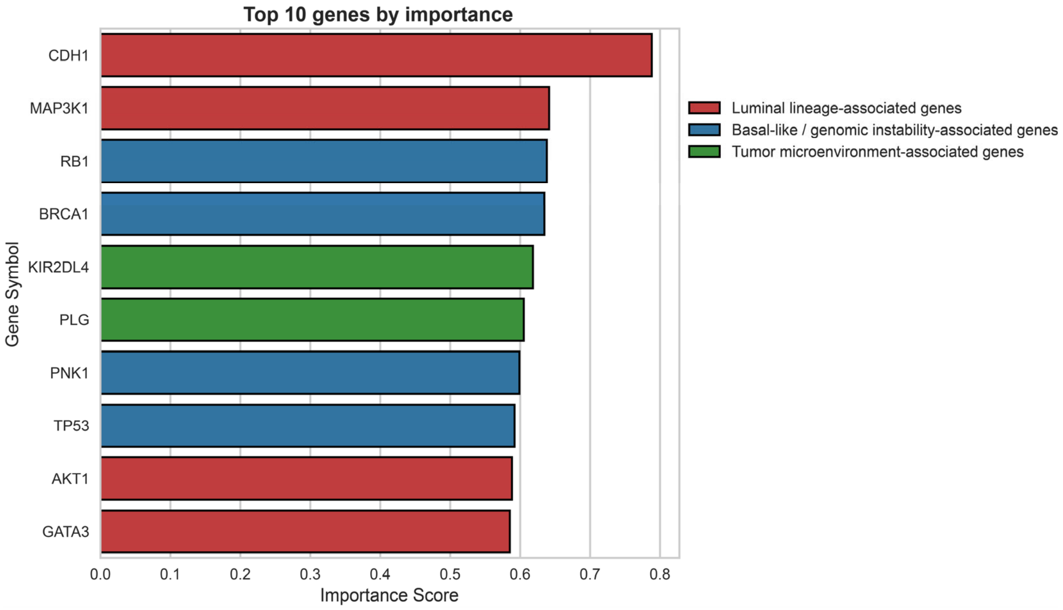

Supplementary Figure S8. Feature importance analysis of deepGene-BC. The top 10 genes ranked by model-derived importance scores are shown, with bars color-coded according to luminal lineage, basal-like/genomic instability, and tumor microenvironment-associated categories.

Supplementary Table S1. Quantitative stability analysis of feature genes identified across varying selection thresholds.

Comparison	Size_A	Size_B	Jaccard	Random Jaccard	Inclusion	Rank Correlation
1% vs 0.5%	71	244	0.22	0.003	0.79	0.99
0.5% vs 0.25%	244	426	0.44	0.008	0.83	0.99
0.25% vs 0.1%	426	626	0.61	0.013	0.94	0.99

Note: The stability of the feature selection process was evaluated by comparing gene sets obtained at four distinct thresholds (1%, 0.5%, 0.25%, and 0.1%). **Comparison:** The specific threshold pairs being compared; **Jaccard:** Jaccard Similarity Coefficient, a measure of similarity between the two sets (Intersection/Union) ; **Random Jaccard:** The expected Jaccard index under random feature selection; **Inclusion**: The proportion of genes from the stricter threshold set that are retained in the looser threshold set (Intersection/Size of Smaller Set). High values indicate strong backward compatibility. **Rank Correlation**: Spearman's rank correlation coefficient (ρ) calculated based on the Mutual Information (MI) scores of the overlapping genes, indicating the consistency of feature prioritization.

Supplementary Table S2. Predictive performance and robustness of deepGene-BC across different feature selection thresholds.

Threshold	Accuracy	Macro-Precision	Macro-Recall	Macro-F1
1%	0.69	0.67	0.67	0.67
0.5%	0.77	0.75	0.75	0.75
0.25%	0.75	0.73	0.72	0.73
0.1%	0.72	0.69	0.70	0.69

Supplementary Table S3. Ablation study of model components of deepGene-BC.

	Wide	FM	Deep	Wide+deep	Wide+deep+FM
Accuracy	0.45	0.65	0.59	0.66	0.77
Macro-Precision	0.44	0.54	0.56	0.62	0.75
Macro-Recall	0.51	0.45	0.58	0.64	0.75
Macro-F1	0.41	0.59	0.53	0.62	0.75

Supplementary Note S1. Model interpretability

As highlighted in the main text, determining whether deepGene-BC's superior performance stems from biologically meaningful patterns rather than spurious correlations is critical for clinical translation. The deepGene-BC model utilizes a hybrid architecture that integrates linear, factorization machine (FM), and deep nonlinear components to process sparse somatic mutation profiles. While this design enables the capture of complex, high-order feature interactions, it inherently functions as a "black box." In an effort to decode this complexity and gain insights into the model's decision-making logic, we conducted a comprehensive post-hoc feature importance analysis. Specifically, we quantified the contribution of each gene by aggregating its weights from both the linear (independent effect) and embedding (interaction potential) components, allowing us to identify the key genomic drivers underlying the subtype predictions. Specifically, for each gene, we computed a gene-level importance score by aggregating the L2 norm of its linear weights with the L2 norm of its embedding vector, which is shared by both the FM and Deep components. Representing the overall contribution of each gene to the predicted probabilities across all breast cancer subtypes.

The top 10 features identified by the model (**Supplementary Figure S8**) demonstrate a high degree of concordance with the established molecular taxonomy of breast cancer. Specifically, the model prioritized *CDH1, GATA3, MAP3K1*, and *AKT1*, which are closely associated with luminal lineage differentiation and hormone signaling [1–3]. Additionally, it identified *TP53, BRCA1, RB1*, and *PNK1*, which are key regulators of genomic instability and DNA damage response and are characteristic of more aggressive Basal-like tumors [2,4,5]. Furthermore, the selection includes *KIR2DL4* and *PLG*, which are linked to tumor microenvironment-associated processes [6,7]. While simple, the biological coherence of these top-ranked features suggests that the model's performance is driven by sound mechanisms rather than random noise, providing preliminary support for the interpretability of deepGene-BC.

References

1. Asselin-Labat, M.-L.; Sutherland, K.D.; Barker, H.; Thomas, R.; Shackleton, M.; Forrest, N.C.; Hartley, L.; Robb, L.; Grosveld, F.G.; van der Wees, J.; et al. Gata-3 is an ess ential regulator of mammary-gland morphogenesis and luminal-cell differentiation. *Natur e Cell Biology* **2007**, *9*, 201–209, doi:10.1038/ncb1530.
2. Ciriello, G.; Gatza, Michael L.; Beck, Andrew H.; Wilkerson, Matthew D.; Rhie, Suh n K.; Pastore, A.; Zhang, H.; McLellan, M.; Yau, C.; Kandoth, C.; et al. Comprehensiv e Molecular Portraits of Invasive Lobular Breast Cancer. *Cell* **2015**, *163*, 506–519, doi: 10.1016/j.cell.2015.09.033.
3. Ellis, M.J.; Ding, L.; Shen, D.; Luo, J.; Suman, V.J.; Wallis, J.W.; Van Tine, B.A.; Hoo g, J.; Goiffon, R.J.; Goldstein, T.C.; et al. Whole-genome analysis informs breast cance r response to aromatase inhibition. *Nature* **2012**, *486*, 353–360, doi:10.1038/nature1114 3.
4. Hoadley, K.A.; Yau, C.; Wolf, D.M.; Cherniack, A.D.; Tamborero, D.; Ng, S.; Leiserson, M.D.M.; Niu, B.; McLellan, M.D.; Uzunangelov, V.; et al. Multiplatform Analysis of 12 C

ancer Types Reveals Molecular Classification within and across Tissues of Origin. *Cell* **2014**, *158*, 929–944, doi:10.1016/j.cell.2014.06.049.
5. Lord, C.J.; Ashworth, A. BRCAness revisited. *Nat. Rev. Cancer* **2016**, *16*, 110–120, doi:10.1038/nrc.2015.21.
6. Duffy, M.J.; McGowan, P.M.; Harbeck, N.; Thomssen, C.; Schmitt, M. uPA and PAI-1 as biomarkers in breast cancer: validated for clinical use in level-of-evidence-1 studies. *Breast Cancer Research* **2014**, *16*, 428, doi:10.1186/s13058-014-0428-4.
7. Long, E.; Kim, H.; Liu, D.; Peterson, M.; Rajagopalan, S. Controlling Natural Killer Cell Responses: Integration of Signals for Activation and Inhibition. In *Annual Review of Immunology, Vol 31*, Littman, D.R., Yokoyama, W.M., Eds.; Annual Review of Immunology; Annual Reviews: Palo Alto, 2013; Volume 31, pp. 227–258.
